# High Clinical Burden of *Cryptosporidium* spp. in Adult Patients with Acquired Immunodeficiency in Ghana

**DOI:** 10.3390/microorganisms12112151

**Published:** 2024-10-26

**Authors:** Fred Stephen Sarfo, Hagen Frickmann, Albert Dompreh, Shadrack Osei Asibey, Richard Boateng, Felix Weinreich, Edmund Osei Kuffour, Betty Roberta Norman, Veronica Di Cristanziano, Torsten Feldt, Kirsten Alexandra Eberhardt

**Affiliations:** 1 Department of Medicine, Komfo Anokye Teaching Hospital, Kumasi 00233, Ghanabranorman@yahoo.com (B.R.N.); 2 Department of Medicine, Kwame Nkrumah University of Science and Technology, Kumasi 00233, Ghana; 3 Department of Microbiology and Hospital Hygiene, Bundeswehr Hospital Hamburg, 20359 Hamburg, Germany; frickmann@bnitm.de; 4 Institute for Medical Microbiology, Virology and Hygiene, University Medicine Rostock, 18057 Rostock, Germany; 5 Department of Clinical Microbiology, Komfo Anokye Teaching Hospital, Kumasi 00233, Ghana; 6 1 German-Netherlands Corps, 48143 Münster, Germany; felixweinreich@bundeswehr.org; 7 Laboratory of Retrovirology, The Rockefeller University, New York, NY 10065, USA; 8 Institute of Virology, Faculty of Medicine and University Hospital Cologne, University of Cologne, 50937 Cologne, Germany; 9 Clinic of Gastroenterology, Hepatology and Infectious Diseases, University Hospital Düsseldorf, 40225 Düsseldorf, Germany; 10 Department of Tropical Medicine, Bernhard Nocht Institute for Tropical Medicine and I. Department of Medicine, University Medical Center Hamburg-Eppendorf, 20359 Hamburg, Germany

**Keywords:** HIV, cryptosporidiosis, parasite, sub-Sahara, enteric infection, diarrhea, Africa, epidemiology, protozoa

## Abstract

There is a paucity of information on the prevalence, risk factors, and clinical correlates of people living with HIV (PLWH) who are co-infected with *Cryptosporidium* spp. in the post-combined antiretroviral therapy era in Ghana. To provide such data, in this observational study, stool samples of 640 HIV-positive and 83 HIV-negative individuals in Ghana were screened for *Cryptosporidium* spp. Additionally, sociodemographic parameters, clinical symptoms, medication intake, and immunological parameters were assessed. The prevalence of *Cryptosporidium* spp. was 11.8% (n = 73) in HIV-positive and 1.2% (n = 1) in HIV-negative participants (*p* < 0.001). Within the group of HIV-positive participants, the prevalence reached 26.0% in patients with CD4+ T cell counts below 200 cells/µL and 46.2% in the subgroup with CD4+ T cell counts below 50 cells/µL. The frequencies of the clinical manifestation of weight loss and gastrointestinal symptoms were significantly higher in patients with *Cryptosporidium* spp. compared to those without co-infection (45.8% vs. 21.4%, *p* < 0.001 and 22.2% vs. 12.2%, *p* = 0.031, respectively). In the modern post-cART era, the acquisition of *Cryptosporidium* spp. among PLWH in Ghana is driven largely by the degree of immunosuppression. Access to cART and screening for *Cryptosporidium* spp. as part of routine care might help control and reduce the burden of the infection.

## 1. Introduction

*Cryptosporidium* spp. are intracellular protozoan parasites which infect the gastrointestinal tract of humans causing diarrheal disease. Of the 38 species of *Cryptosporidium* spp. recognized, *Cryptosporidium parvum* and *Cryptosporidium hominis* are responsible for the majority of human infections [1]. The mode of transmission is via the fecal–oral route through the consumption of contaminated food and water as well as contact with infected individuals or animals [2]. Infection in immunocompetent persons is either asymptomatic or manifests with profuse watery diarrhea associated with nausea, vomiting, stomach cramps, and, occasionally, fever for approximately two weeks [3]. However, among immunocompromised patients, such as people living with HIV (PLWH), transplant and chemotherapy recipients, and patients undergoing treatment for hemodialysis and cancer, infection with *Cryptosporidium* spp. causes prolonged symptoms culminating in chronic diarrhea lasting two or more months or fulminant diarrhea with the passage of >2 L of watery stools per day [2]. *Cryptosporidium* spp. are estimated to be responsible for more than 8 million foodborne illness cases worldwide annually, and, to date, there is no vaccine available to protect against cryptosporidiosis [4]. Nitazoxanide, the only FDA-approved drug to treat cryptosporidiosis, has only a very limited efficacy in immunocompetent individuals and is mostly ineffective in HIV-positive patients [5,6].

Cryptosporidiosis is an AIDS-defining event and was a major cause of mortality in the wake of the HIV/AIDS epidemic [7]. A systematic review conducted in the year 2020 involving 51,123 HIV-infected patients from 161 studies reported an overall prevalence of 11.2% (95% confidence interval: 9.4, 13.0%) [8]. The prevalence of *Cryptosporidium* spp. among PLWH varied depending on the diagnostic approach used for the study, ranging from 10.0% (8.4–11.8%) using staining methods to over 13.5% (8.9–19.8%) using molecular methods and up to 26.3% (15.0–42.0%) using antigen detection methods, each being affected by method-intrinsic diagnostic accuracy limitations. The identified risk factors for suffering from *Cryptosporidium* spp. infection among PLWH include having diarrhea, being naïve to antiretroviral therapy, and having a CD4+ T cell count below 200 cells/mm^3^ [8]. A recent meta-analysis from Ethiopia documented a reduction in the prevalence of *Cryptosporidium* spp. among PLWH over the past two decades from 14.6% in the early 2000s to 6.7% in 2019 largely due to early diagnosis of HIV and prompt initiation of potent combination antiretroviral therapy (cART) [9]. The prevalence of *Cryptosporidium* spp., its risk factors, and clinical correlates in the post-ART era have seldom been reported in Ghana, a West African country. We therefore sought to deploy molecular methodology to characterize the prevalence, risk factors, and correlates of recorded cycling threshold values of *Cryptosporidium* spp. among PLWH, stratified by cART status, in a tertiary medical center in the middle belt of Ghana.

## 2. Materials and Methods

### 2.1. Study Design and Population

This observational study is part of a work investigating the associations of gastrointestinal and other pathogens with immunological parameters in HIV-positive and HIV-negative adults in Ghana [10]. Over a period of 12 months, consecutive HIV-positive patients at the HIV outpatient department of the Komfo Anokye Teaching Hospital and an HIV-negative control group were offered to participate in the study. All participants provided written informed consent prior to enrolment.

### 2.2. Data Collection and Laboratory Methods

Demographic and clinical data were assessed using a standardized questionnaire. Blood samples were collected, and the analysis of CD4+ T cell counts was performed locally using a FACSCalibur flow cytometer (Becton Dickinson, Mountain View, CA, USA). HIV-1 viral load was measured using the Real-Time HIV-1 PCR system (Abbott Diagnostics, Wiesbaden, Germany).

Aliquots of stool samples were freshly frozen and stored at −80 °C. Nucleic acids were extracted applying the QIAamp stool DNA mini kit (Qiagen, Hilden, Germany) in line with the manufacturer’s protocols. Subsequently, the eluates were once more stored at −80 °C until PCR analysis was performed. Real-time in-house PCR for *Cryptosporidium* spp. targeted a 159-base pair sequence of the small subunit ribosomal RNA (SSU rRNA) gene as described recently, for which a sensitivity of virtually 100% and a specificity of 96.9% had been calculated and a limit of detection of <10 copies per µL of eluate had been shown in recent assessments [11,12]. The in-house assay was run on magnetic induction cyclers (MIC, Bio Molecular Systems Ltd., London, UK) in 20 µL volumes. In short, the applied oligonucleotides comprised the forward primer JVAF 5′-ATGACGGGTAACGGGGAAT-3′, the reverse primer JVAR 5′-CCAATTACAAAACCAAAAAGTCC-3′, and the hybridization probe JVAP18S 5′-CY3-CGCGCCTGCTGCCTTCCTTAGATG-BHQ2-3′. The PCR reaction mix was based upon of the HotStarTaq Mastermix (Qiagen, Hilden, Germany) with a final Mg^2+^ concentration of 5 mM. The oligonucleotide concentrations were 250 nM for the primers and 300 nM for the probe. In each PCR run, a PCR grade water-based negative control as well as a positive control based on a plasmid with the inserted *Cryptosporidium* spp. sequence 5′-TACCGTGGCAATGACGGGTAACGGGGAATTAGGGTTCGATTCCGGAGAGGGAGCCTGAGAAACGGCTACCACATCTAAGGAAGGCAGCAGGCGCGCAAATTACCCAATCCTAATACAGGGAGGTAGTGACAAGAAATAACAATACAGGACTTTTTGGTTTTGTAATTGGAATGAGTTAA-3′ (NCBI GenBank accession number AY458612) in a pEX-A128 vector backbone were included. Sample inhibition was assessed by applying a phocid herpes virus DNA-specific real-time PCR as previously published [13]. The reaction profile of the PCR assay comprised an initial denaturation at 95 °C for 15 min followed by 45 cycles of denaturation for 15 s at 95 °C and annealing as well as amplification for 60 s at 60 °C. Subsequently, the reaction mix was cooled down to 40 °C for an additional 20 s.

### 2.3. Statistical Analysis

The continuous variables were expressed as median (interquartile range, IQR) or mean ± standard deviation (SD) and compared using the Wilcoxon rank sum test or the unpaired Student’s *t*-test. The categorical variables were compared using either the χ^2^ test or the Fisher exact test, as appropriate. Multiple logistic regression analysis was performed using the ‘forestmodel’ package in R (version 4.0.5, R Foundation for Statistical Computing, Vienna, Austria). The Spearman rank correlation coefficient ρ was calculated as a measure of strength of the relationship between continuous variables. Two-sided *p*-values were presented, and an α of 0.05 was considered as statistically significant.

## 3. Results

### 3.1. Composition of the Study Population

**Figure 1:**
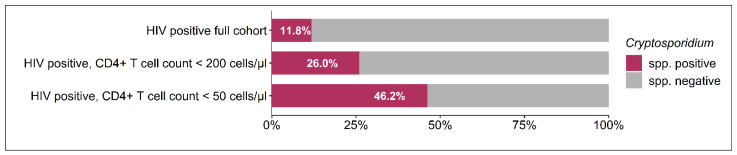
Prevalence of *Cryptosporidium* spp. in HIV-positive participants according to the CD4+ T cell count.

A total of 1202 individuals (1095 HIV-positive and 107 HIV-negative) were included in this study. Residual stool samples for *Cryptosporidium* spp. screening were available for 640 HIV-positive and 83 HIV-negative persons, of which 22 samples had to be excluded from further assessment because of the recorded PCR inhibition. The prevalence of *Cryptosporidium* spp. was 11.81% (n = 73) in HIV-positive patients and 1.20% (n = 1, *p* = 0.001) in HIV-negative participants. Figure 1 demonstrates that more than every fourth patient (25.97%) with a CD4+ T cell count below 200 cells/µL and almost every second patient (46.15%) in the subgroup of PLWH with a CD4+ T cell count below 50 cells/µL were co-infected with *Cryptosporidium* spp. In the group of HIV-positive individuals with CD4+ T cell counts above 200 cells/µL, the prevalence of *Cryptosporidium* spp. was 6.01% (n = 25) and not found to be significantly different from the prevalence that was detected in the HIV-negative control group (*p* = 0.10).

### 3.2. Comparison of Demographic and Clinical Characteristics of the HIV Cohort According to Cryptosporidium spp. Status

HIV-positive participants with or without colonization of *Cryptosporidium* spp. were not different regarding their age or sex (Table 1). Furthermore, no differences in the proportion of patients with access to tap water (54.17% vs. 52.63%) or any of the other recorded socioeconomic parameters were detected. HIV-positive patients co-infected with *Cryptosporidium* spp. were more often diagnosed with HIV within the last month (66.15% vs. 34.02%, *p* < 0.001) and less often treated with cART (15.28% vs. 45.30%, *p* < 0.001). In patients already treated with cART, no significant difference was found in the timespan since treatment initiation. When stratifying for CD4+ T cell count, also in the subgroup of HIV-positive patients with counts above 200/µL, the prevalence of *Cryptosporidium* spp. was higher in cART-naïve participants compared to those exposed to cART (8.99% vs. 3.52%, *p* = 0.033). In the subgroup of PLWH with CD4+ T cell counts below 200 µ/L, prevalences could not be shown to be statistically different in cART-naïve versus cART-exposed individuals (28.03% vs. 12.50%, *p* = 0.136).

**Figure 2:**
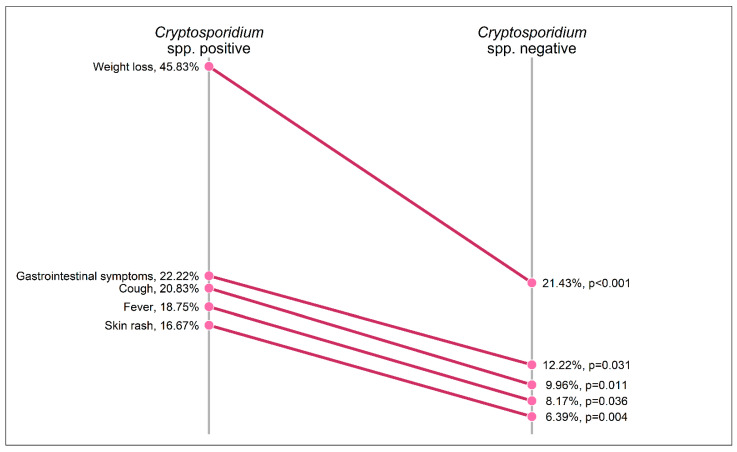
Prevalence of clinical symptoms during the last six months according to *Cryptosporidium* spp. status among HIV-positive participants.

HIV-positive patients co-infected with *Cryptosporidium* spp. reported the presence of clinical symptoms at a significantly higher frequency than HIV-positive participants without *Cryptosporidium* spp. infection (Figure 2). In detail, 45.83% of co-infected patients reported the presence of weight loss during the last six months (vs. 21.43% in HIV-positive *Cryptosporidium* spp.-negative participants, *p* < 0.001), 22.22% suffered from gastrointestinal symptoms (vs. 12.22%, *p* = 0.031), 20.83% had an acute or chronic cough (vs. 9.96%, *p* = 0.011), 18.75% experienced a fever during the last six months (vs. 8.17%, *p* = 0.036), and 16.67% reported the occurrence of a skin rash (vs. 6.39% in *Cryptosporidium* spp.-negative PLWH, *p* = 0.004). Within the group of documented gastrointestinal symptoms, acute diarrhea and abdominal pain were more frequent in patients with the presence of *Cryptosporidium* spp. (12.50% vs. 5.26%, *p* = 0.032 and 12.50% vs. 6.02%, *p* = 0.071, respectively), while the occurrence of nausea or vomiting was reported at a similar frequency in both groups (6.94% vs. 3.95%, *p* = 0.386).

### 3.3. Comparison of Virological and Immunological Characteristics of the HIV Cohort According to Cryptosporidium spp. Status

PLWH receiving cART with *Cryptosporidium* spp. co-infection had a significantly higher median HIV-1 viral load in log10 copies/mL compared to those without this co-infection (3.4 [2.1–4.7 IQR] vs. 1.6 [0.0–1.8 IQR], *p* = 0.001, Table 2). Also, in cART-naïve patients, HIV-1 viral loads were significantly higher in *Cryptosporidium* spp.-positive participants (5.5 [5.0–5.8 IQR] vs. 5.0 [4.2–5.5 IQR], *p* < 0.001). Furthermore, lower CD4+ T cell counts/µL were found in cART-naïve patients with *Cryptosporidium* spp. colonization compared to those patients without detection of this pathogen (76 [30–217 IQR] vs. 266 [122–466 IQR], *p* < 0.001). Whereas no differences in CD8+ T cell counts were detected, the CD4+/CD8+ T cell ratio, which is inversely associated with immune activation in HIV, was significantly lower in *Cryptosporidium* spp. carriers in cART-exposed individuals (0.2 [0.1–0.5 IQR] vs. 0.5 [0.4–0.9 IQR], *p* = 0.014), as well as in cART-naïve patients (0.1 [0.0–0.2 IQR] vs. 0.3 [0.1–0.5 IQR], *p* < 0.001), compared to PLWH without this co-infection.

### 3.4. Factors Associated with the Reporting of Weight Loss During the Last Six Months in HIV-Positive Participants

**Figure 3:**
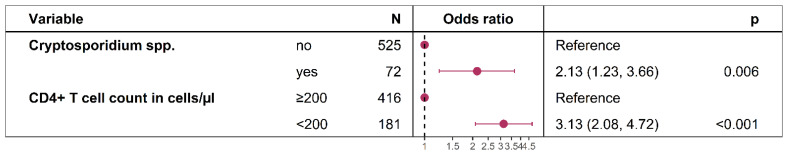
Logistic regression model: Factors associated with weight loss during the last 6 months.

Figure 3 demonstrates that *Cryptosporidium* spp. status was independently associated with weight loss during the last 6 months in HIV-positive participants after adjusting for the CD4+ T cell count as a covariate.

### 3.5. Correlations of Cycle Threshold (Ct) Values with HIV Viral Load and CD4+ Cell Count

The correlation analysis of *Cryptosporidium* spp.-specific cycle threshold (Ct) values in real-time PCR and HIV viral load as well as CD4+ T cell count in *Cryptosporidium* spp.-positive participants revealed a significant correlation between the CD4+ lymphocyte count and the Ct values of *Cryptosporidium* spp. and an inverse correlation between the HIV viral load and the Ct values of *Cryptosporidium* spp. (rho = 0.49, *p* = <0.001 and rho = −2.4, *p* = 0.045, respectively, Table 3).

## 4. Discussion

Intestinal parasite infections caused by protozoa are common in PLWH in developing countries [14]. In this large cohort of Ghanaians living with HIV in the post-cART era, the prevalence of *Cryptosporidium* spp. was 11.81%, and it was 1.20% among an HIV-negative control group. Compared with data from other African countries, the prevalence of *Cryptosporidium* spp. in PLWH in Ghana was lower than the 34.9% in South Africa [15], as high as in Ethiopia (11%) [9], and higher than the 9.5% in Zambia [16]. These different prevalences might be explained by differences in the relative proportions of cART exposure in HIV-positive cohorts or the diagnostic techniques utilized [3,17]. In the present study, the presence of *Cryptosporidium* spp. was assessed using an in-house real-time PCR targeting the small subunit ribosomal RNA (SSU rRNA) gene, for which a sensitivity of virtually 100% and a specificity of 96.9% had been calculated and a limit of detection of <10 copies per µL of eluate had been shown in previous assessments [11,12]. The low prevalence of *Cryptosporidium* spp. in HIV-negative participants is in accordance with the 1% in a healthy control group from a recent study assessing the prevalence of enteric protozoans in persons with diabetes mellitus at the same hospital in Ghana [18].

In our cohort, the prevalence of *Cryptosporidium* spp. co-infection was higher among PLWH with CD4+ T cell counts below 200 cells/µL (25.97%) and in the subgroup with CD4+ T cell counts below 50 cells/µL (46.15%). In the group of HIV-positive individuals with CD4+ T cell counts above 200 cells/µL, the prevalence of *Cryptosporidium* spp. was 6.01% and not found to be significantly different from the prevalence that was detected in the HIV-negative control group. Furthermore, the HIV-1 viral load and the CD4+/CD8+ T cell ratio, as a marker for immune activation, were found to be unfavorable in patients co-infected with *Cryptosporidium* spp. These observations strongly indicate that this co-infection is correlated with the level of immune suppression of the host and are also consistent with a meta-analysis from Ethiopia indicating that HIV-infected people with a low CD4+ T cell count (CD4 < 200 cells/mm^3^) were 13.07 times more likely to become persistently infected with *Cryptosporidium* spp. than those with a high CD4+ T cell count (CD4 > 500 cells/mm^3^) (OR: 13.07 (95%CI: 6.38–26.75)) [9]. The higher prevalence of *Cryptosporidium* spp. associated with a lower CD4+ T cell count might be due to deprivation of the immune cells that make the patients more vulnerable to getting infected with particular parasites and unable to clear them once the infection is established [19]. Interestingly, the above-mentioned meta-analysis showed a declining trend in the prevalence of *Cryptosporidium* spp. over the last two decades. *Cryptosporidium* spp. infection in PLWH in Ethiopia decreased in abundance in 2001–2010, 2011–2014, and 2015–2019, with prevalences of 14.6% (95%CI: 0.076–0.217), 12.71% (95%CI: 0.086–0.167), and 6.7% (95%CI: 0.044–0.090), respectively. This decrease could be linked to the reduced prevalence of new HIV cases, the improved use of antiretroviral treatment, improved sanitary practices, and access to safe potable water [9]. In line with this, the present work suggests a decrease in the prevalence of *Cryptosporidium* spp. in PLWH, which was found to be 14% in 2008 [20] and 11.8% in 2012 at the same hospital. However, due to different detection methods, a direct comparison of these studies is not possible. Furthermore, there is no information on the proportion of patients receiving cART or their immune status in the study by Boaitey and colleagues [20].

Another observation made in the present work is that *Cryptosporidium* spp.-specific cycle threshold values in real-time PCR correlate with CD4+ T cell counts and inversely correlate with HIV-1 viral loads. This might demonstrate that not only the presence or absence of *Cryptosporidium* spp. is associated with the immune status of individuals but also the quantitative load of this pathogen.

Importantly, PLWH co-infected with *Cryptosporidium* spp. suffered significantly more frequently from clinical symptoms, namely weight loss (45.83%), gastrointestinal symptoms (22.22%), an acute or chronic cough (20.83), a fever during the last six months (18.75%), and the occurrence of a skin rash (16.67%). While these symptoms might also be a direct or indirect consequence of the impaired immune system rather than being linked to a gastrointestinal infection, a multiple logistic regression model revealed an independent association of weight loss during the last 6 months in HIV-positive participants and the presence of *Cryptosporidium* spp. after adjusting for the CD4+ T cell count. These findings are different from those in a recent study assessing *Cryptosporidium* spp. co-infections in HIV-positive patients in the Central Region of Ghana [21], which, however, used a different wet lab methodology. In that study, no associations with clinicals symptoms were detected. However, the body of literature is consistent with our finding of a high clinical burden of PLWH co-infected with *Cryptosporidium* spp. [2,22,23].

In contrast to some other studies, we did not observe differences in socioeconomic factors, such as access to tap water, between *Cryptosporidium* spp.-positive and -negative PLWH [16,21,24]. A potential explanation might be that patients enrolled in this study were counselled in a standardized way in the outpatient clinic on safeguarding good health through good hygiene practices. Furthermore, our results did not reveal differences in age or sex between the groups, suggesting that these demographic factors were not predetermining factors for this protozoan infection in PLWH [20].

This study has a number of limitations. First, it cannot be excluded that the sample age might have impacted the real-time PCR results due to degradation of target DNA despite appropriate storage at −80 °C. However, as the prevalences found in our study are comparable to those observed in other research works from Africa, the results were classified as plausible. Second, as Ghana is a high-endemicity setting for infectious gastroenteritis, simultaneous colonization with two or more pathogens is common, specifically in immunocompromised patients [25,26]. Therefore, it is difficult to assess if the reported symptoms are associated with *Cryptosporidium* spp. positivity alone or with a combination of gastrointestinal pathogens. Third, although the presence or absence of a range of potential clinical symptoms were recorded for study participants, more specific details on single symptoms, such as the duration in days, the frequency of diarrhea per day, or the color and consistency of diarrhea, were not documented for each participant. However, as these details might give more insight into the clinical burden than the presence or absence of symptoms alone, recording and analyzing these additional details for each symptom might be considered in future studies. Fourth, the cross-sectional study design does not allow for the drawing of causal inferences concerning the described associations.

## 5. Conclusions

*Cryptosporidium* spp. infection remains a major cause of morbidity in HIV-infected people in Ghana, especially those with a suboptimal CD4+ T cell count. Thus, routine screening for *Cryptosporidium* spp. in PLWH should be incorporated as part of routine care to enable early diagnosis and prompt treatment, coupled with improved hygienic practices and access to clean potable water. Furthermore, access to cART with subsequent suppressed HIV viral loads remains one of the main goals in controlling and reducing the high burden of infection with *Cryptosporidium* spp.

## Figures and Tables

**Table 1:** Demographics, socioeconomic parameters, medical treatment, and virological and immunological parameters in HIV-infected individuals according to *Cryptosporidium* spp. status.

	Variable	HIV Positive *Cryptosporidium* spp. Positive, n = 73 (11.81%)	HIV Positive *Cryptosporidium* spp. Negative, n = 545 (88.19%)	*p*-Value
Demographics	Age in years ± SD	41.0 ± 10.7	40.4 ±9.3	0.656
	Female, n (%)	50 (69.44)	400 (75.19)	0.365
Socioeconomic parameters	Access to tap water, n (%)	39 (54.17)	280 (52.63)	0.905
	Electricity in household, n (%)	68 (94.44)	496 (93.23)	1.000
	Television in household, n (%)	59 (81.94)	434 (81.58)	1.000
	Refrigerator in household, n (%)	57 (79.17)	381 (71.62)	0.228
	Owning a car, n (%)	8 (11.11)	51 (9.59)	0.843
Medical therapy	HIV diagnosis since < 1 month, n (%)	43 (66.15)	164 (34.02)	<0.001
	TMP/SMX prophylaxis, n (%)	29 (40.85)	165 (31.67)	0.158
	Intake of cART, n (%)	11 (15.28)	241 (45.30)	<0.001
	Months since initiation of cART, median (IQR)	60.9 (37.2 to 71.9)	50.9 (23.8 to 79.3)	0.537

SD—standard deviation; TMP/SMX—Trimethoprim/sulfamethoxazole; IQR—Interquartile range.

**Table 2:** Virological and immunological parameters according to *Cryptosporidium* spp. and ART status.

Variable	HIV Positive cART Exposed			HIV Positive cART Naïve		
	*Cryptosporidium* spp. Positive, Median (IQR), n = 11 (4.37%)	*Cryptosporidium* spp. Negative, Median (IQR), n = 241 (95.63%)	*p*-Value	*Cryptosporidium* spp. Positive, Median (IQR), n = 61 (17.33%)	*Cryptosporidium* spp. Negative, Median (IQR), n = 291 (82.67)	*p*-Value
Viral load, log10 copies/mL	3.4 (2.1–4.7)	1.6 (0.0–1.8)	0.001	5.5 (5.0–5.8)	5.0 (4.2–5.5)	<0.001
CD4+ T cell count/µL	345.0 (180.0–654.5)	503.0 (312.8–702.5)	0.225	76.0 (30.0–217.0)	266.0 (112.0–466.0)	<0.001
CD8+ T cell count/µL	967.5 (726.0–1584.5)	918.0 (652.5–1288.5)	0.536	927.0 (523.0–1328.5)	1022.5 (672.2–1573.5)	0.273
CD4+/CD8+ T cell ratio	0.2 (0.1–0.5)	0.5 (0.4–0.9)	0.014	0.1 (0.0–0.2)	0.3 (0.1–0.5)	<0.001

IQR—Interquartile range.

**Table 3:** Correlation of cycle threshold (Ct) values of the real-time PCR targeting *Cryptosporidium* spp. with HIV viral load and CD4+ T cell count.

	Ct Values of the Real-Time PCR Targeting *Cryptosporidium* spp.	
	Spearman’s Rho	*p*-Value
CD4+ T cell count/µL	0.488	<0.001
Viral load, log10 copies/mL	−0.244	0.045

## Data Availability

All relevant data are provided in the manuscript. Raw data can be made available upon reasonable request.
